# Nano(bio)Materials Do Not Affect Macrophage Phenotype—A Study Conducted by the REFINE Project

**DOI:** 10.3390/ijms25105491

**Published:** 2024-05-17

**Authors:** Christopher A. W. David, Jolanda P. Vermeulen, Sabrina Gioria, Rob J. Vandebriel, Neill J. Liptrott

**Affiliations:** 1 Immunocompatibility Group, Department of Pharmacology and Therapeutics, Institute of Systems, Molecular and Integrative Biology, University of Liverpool, Liverpool L7 3NY, UK; cdavid@liverpool.ac.uk; 2 Centre of Excellence for Long-Acting Therapeutics (CELT), Institute of Systems, Molecular and Integrative Biology, University of Liverpool, Liverpool L7 8TX, UK; 3 National Institute for Public Health & the Environment, 3720 BA Bilthoven, The Netherlands; jolanda.vermeulen@rivm.nl (J.P.V.); rob.vandebriel@rivm.nl (R.J.V.); 4 European Commission, Joint Research Centre (JRC), 21027 Ispra, Italy; sabrina.gioria@ec.europa.eu

**Keywords:** monocyte-derived macrophages, nanostructured lipid carrier, polymeric nanomedicine, immunotoxicity assessment

## Abstract

Macrophages are well known for their involvement in the biocompatibility, as well as biodistribution, of nano(bio)materials. Although there are a number of rodent cell lines, they may not fully recapitulate primary cell responses, particularly those of human cells. Isolation of tissue-resident macrophages from humans is difficult and may result in insufficient cells with which to determine the possible interaction with nano(bio)materials. Isolation of primary human monocytes and differentiation to monocyte-derived macrophages may provide a useful tool with which to further study these interactions. To that end, we developed a standard operating procedure for this differentiation, as part of the Regulatory Science Framework for Nano(bio)material-based Medical Products and Devices (REFINE) project, and used it to measure the secretion of bioactive molecules from M1 and M2 differentiated monocytes in response to model nano(bio)materials, following an initial assessment of pyrogenic contamination, which may confound potential observations. The SOP was deployed in two partner institutions with broadly similar results. The work presented here shows the utility of this assay but highlights the relevance of donor variability in responses to nano(bio)materials. Whilst donor variability can provide some logistical challenges to the application of such assays, this variability is much closer to the heterogeneous cells that are present in vivo, compared to homogeneous non-human cell lines.

## 1. Introduction

Macrophages are tissue-resident innate immune cells that, depending on cues from their microenvironment, play a wide variety of roles [1]. Their activation states have been characterised, broadly, as either M1 (classically activated) or M2 (alternatively activated) [2]. These subtypes are discriminated by phenotypic and functional differences that result in distinct roles for each. Broadly, M1 macrophages produce pro-inflammatory cytokines that combat pathogenic infection and reduce the infectivity of microbes, while M2 macrophages produce growth factors and anti-inflammatory cytokines to suppress the host immune response, promote wound healing and tissue remodelling, and improve metabolic and endocrine signalling within tissues [3,4].

Macrophages are well known to uptake nanoparticles and nano(bio)materials (NBMs), as a mechanism to clear them from the body [5], and to be involved in tolerance to regular exposure to particulate matter [6]. Following opsonisation, by opsonins such as antibodies and complement proteins, nanoparticles are taken up into cells of the mononuclear phagocyte system via liver Kupffer cells and splenic macrophages, for clearance from the body [7,8]. However, it has been described that accumulation and persistence in macrophages may contribute to ongoing inflammation [5] as well as cell-mediators of complement-related hypersensitivity [9]. In addition to their involvement as sentinel cells of the immune response, macrophages are also involved in the biodistribution of NBMs following phagocytosis, where the foreign substances are isolated via a cell membrane enclosure and degraded by the phagolysosome [10]. Phagocytosis is a receptor-mediated endocytosis specific to phagocytic cells, e.g., cells of the mononuclear phagocytic system [11], and is an active process requiring actin polymerization [12]. There are four main receptors that mediate phagocytic uptake, of which specific physicochemical parameters, including size and surface charge, appear to demonstrate preferential involvement [13,14]. Phagocytosis via three of these receptors (complement receptor, FcγR receptor, and mannose receptor) is accompanied by inflammatory reactions (e.g., cytokine secretion) [15]. Phagocytosis via the fourth receptor (scavenger receptor) is not accompanied by inflammatory responses.

The ability of rodent cell line models to fully mimic the responses of primary human cells to challenge with nano(bio)materials is limited [16,17]. Indeed, the use of human cell lines, such as THP1, as an alternative prove most beneficial in terms of convenience; however, they do not entirely reproduce the responses generated by primary monocyte-derived macrophages (MDMs) [18]. Given their importance in addressing both the biocompatibility and biodistribution of nanoparticles, it is evident that a robust methodology for generating macrophages is required, given the difficulty in obtaining sufficient human cells for such exploratory work. In-vitro-polarised MDMs provide a useful model to investigate the uptake of NBMs and the subsequent impact they may have on the cells, phenotypically, both functionally and by monitoring secreted factors [19]. However, there are notable differences, and influences, of variation in culture conditions to drive differentiation and polarisation of monocytes towards M1 and M2 phenotypes [20]. With regard to the development of nano(bio)materials, the FDA has released draft guidance for their assessment, which includes assessment of interactions with phagocytic cells, such as macrophages [21].

As part of the Regulatory Science Framework for Nano(bio)material-based Medical Products and Devices (REFINE) project (grant agreement #761104), we sought to define a standardised approach to the isolation of human primary monocytes and differentiation to M1 and M2 phenotypes that proved straightforward and achievable for groups that had limited experience in the space. In the work described here, we deployed a standardised procedure for assessment of both monocyte activation and response of human, polarised, monocyte-derived macrophages (MDMs) to well-characterised NBMs [22]. MDMs have been used previously in assessing the safety of nanomedicines for the treatment of HIV that have subsequently been used in phase I clinical trials (EudraCT, number 2013-004913-41) [23,24], as well as in the development of novel delivery systems [25]. Monocytes mount a response toward pyrogenic material [26] and, therefore, an initial test of possible contamination with pyrogenic material, beyond endotoxins, is essential, as this contamination will influence the development and production of NBMs at stages prior to GMP manufacture, as well as ensuring no false positives in immunotoxicological assessment. Since 2010, the monocyte-activation test (MAT) has been an official test of the European Pharmacopoeia (EP 6.7 chapter 2.6.30) [27], which provides an orthogonal assay to the limulus amoebocyte lysate (LAL) test and an alternative to the rabbit pyrogen test (RPT). Unlike other methods, the MAT does not require the use of animal testing and can also detect responses to pyrogens other than lipopolysaccharides, such as lipoteichoic acid and nucleic acids.

Following determination of pyrogenic potential, in-vitro-polarised MDM of M1 and M2 phenotypes were exposed to test materials, obtained from the REFINE consortium and tested to observe any potential modulation of subtype-specific cytokine secretion, whether activation or inhibition. The work presented here shows the first steps towards a robust protocol for the differentiation of human monocytes for preclinical evaluation of macrophage responses to nano(bio)materials.

## 2. Results

### 2.1. Monocyte-Activation Test

**Figure 1:**
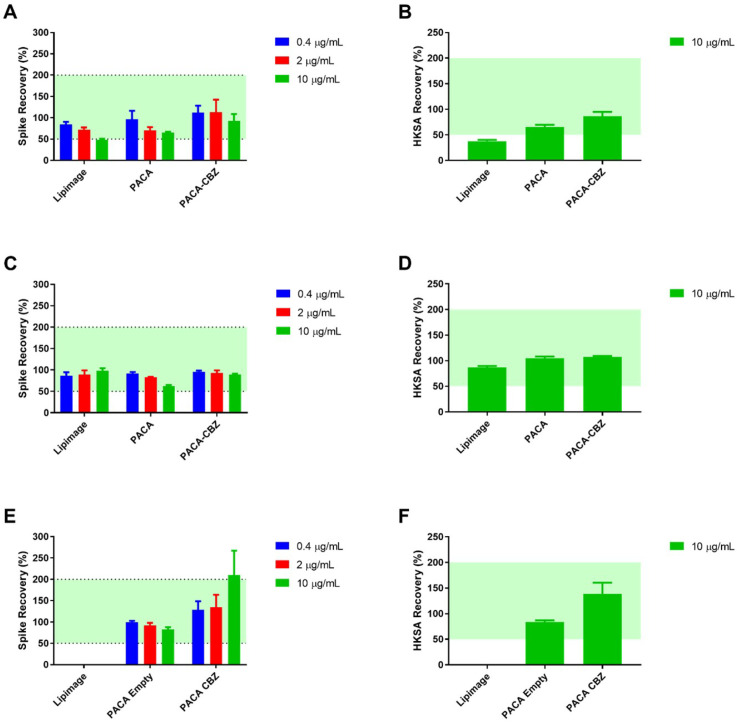
Test for interfering factors: Data from UoL (**A**) PyroDetect^®^ endotoxin standard and (**B**) heat-killed Staphylococcus aureus (HKSA) spike recovery. Data from RIVM (**C**) PyroDetect^®^ endotoxin standard and (**D**) HKSA spike-recovery. Data from JRC (**E**) PyroDetect^®^ endotoxin standard and (**F**) HKSA spike-recovery following incubation with nano(bio)materials, with the exception of Lipimage™ 815, determined via the monocyte activation test. Data displayed as an average of four technical replicates ± standard deviation as a percentage of relevant control. Green shading represents accepted 50–200% recovery range.

The PyroMAT^®^ system uses cryopreserved Mono-Mac-6 human monocytic cells as a source of monocytes. The response to pyrogenic substances is determined by measurement of IL-6 produced by the Mono-Mac-6 cells. None of the nano(bio)materials to be used in subsequent experiments were found to contain any detectable pyrogens, as evidenced by there being no increase in IL-6 production from Mono-Mac-6 cells. Nanoparticles are well known to interfere with biological assays in a number of ways. Testing for interference showed LipImage™ 815, at the highest tested concentration (10 µg/mL), to fall below the accepted 50% limit for endotoxin recovery (48.7%, Figure 1A). The same effect was observed for recovery of the non-endotoxin pyrogen HKSA, at the same material concentration (37.8%, Figure 1B). This underestimation of contamination was alleviated at the greater dilutions of 2 and 0.4 µg/mL, where the recovery percentages were 73.4 and 84.3%, respectively. All tested concentrations of PACA and PACA-CBZ fell within the accepted 50–200% recovery range. The materials were tested identically at additional partner labs (RIVM and JRC), the results of which showed a high degree of similarity, with the exception of the interference by Lipimage not being observed at 10 µg/mL (Figure 1E).

### 2.2. Macrophage Polarisation and Cytokines

**Figure 2:**
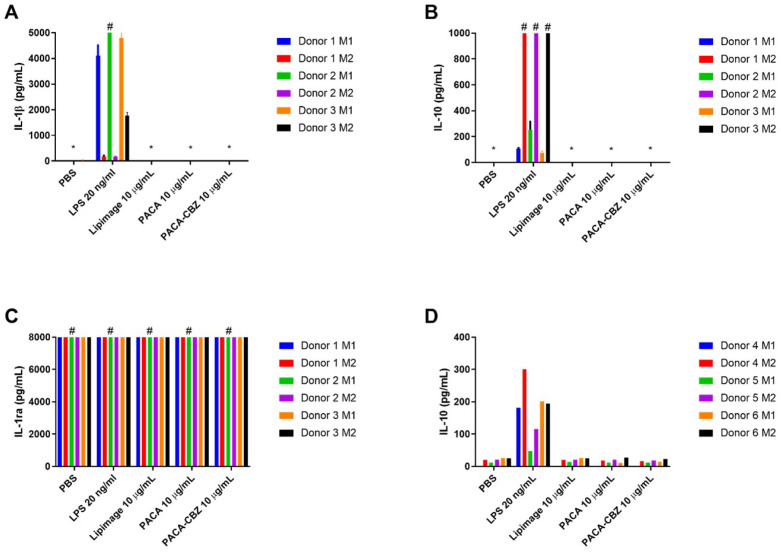
Data from UoL, quantification of cytokines (**A**) IL-1β, (**B**) IL-10, and (**C**) IL-1ra secreted by differentiated M1 and M2 macrophages, from three healthy donors, in response to treatment with negative control (PBS), positive control LPS (20 ng/mL) and nano(bio)materials Lipimage™ 815 (10 µg/mL), PACA (10 µg/mL), and PACA-CBZ (10 µg/mL), determined by multiplex analysis. (**D**) Data from RIVM, quantification by ELISA of IL-10 generated by differentiated M1 and M2 macrophages from an additional three healthy donors following identical treatments. Data displayed as an average of three technical replicates ± standard deviation; * denotes concentrations below the assay limit of detection; # denotes samples exceeding assay limit of quantification.

IL-1β, a cytokine generated in response to pyrogenic stimulation, prototypical of the M1 phenotype, was observed to be generated at a much higher level in LPS-treated M1 macrophages than M2 (Figure 2A). No nano(bio)materials were observed to stimulate IL-1β secretion under the conditions described here. IL-10 secretion by M2 macrophages in response to LPS was observed to be greater than that by M1 phenotype, exceeding the limit of detection for the assay in all three donors (Figure 2B). IL-1ra exceeded the upper limit of quantification for all conditions (Figure 2C). Further dilution to resolve any potential differences would be required but would have resulted in other cytokines, present at lower concentrations, to be undetectable. IL-12 was not stimulated to a quantifiable level by any of the conditions described here. IL-10 secretion by macrophages differentiated from three healthy donors, performed at RIVM, further demonstrate the interindividual variability in response to the defined treatments (Figure 2D). While M2 macrophages of donors 4 and 5 generated more IL-10 than their respective M1 in response to LPS, the concentrations are markedly less than those of donors 1, 2, and 3. Beyond this, the concentration of IL-10, in response to LPS, by both M1 and M2 of donor 6 are comparable, and all other treatments generated a low but quantifiable concentration of this cytokine.

## 3. Discussion

It has been highlighted that existing methodologies, when applied to nanoparticles and NBMs, are not always sufficient to observe and characterise the complex interactions that may occur [28]. It is for this reason that outputs of these tests must be re-evaluated and reinterpreted, such that interactions should be evaluated in a broader context. Furthermore, development and establishment of nanoparticle- and NBM-specific testing will prove to be essential as more complex nano(bio)materials are produced. The assessments described in this work have been highlighted among others that, currently, are a methodological requirement in the quality and safety characterisation of nanotechnology-based health products [28,29].

Nano(bio)materials, depending on their chemistry or loaded compounds, have the potential to interact with biological tests in a manner that, if not detected and accounted for, would lead to over/under estimation of their biological impact [30]. The MAT, applied to this set of NBMs, follows established criteria where the validity of endotoxin-spiked samples is accepted when recovery falls within the range of 50–200%, beyond which further dilutions of the relevant material should be tested to determine if this was a result of concentration or material-dependent effects [31,32]. While not developed specifically with NBMs in mind, this forward-thinking approach is highly useful for acknowledging and/or circumventing the well-known issue of nanoparticle interference, with this and related assays such as the Limulus amoebocyte lysate (LAL) assay [33,34].

The cell-based platform of the MAT provides additional benefit over other methods for detecting endotoxin contamination, such as LAL, by assessing the response to other potential pyrogens (e.g., lipoteichoic acid, nucleic acids) that would otherwise not be detected by LAL assessment. Additionally, the MAT shows the level of biological response to a known number of pyrogens, enabling the determination of effect levels. In addition to the quantified IL-6, endotoxin upregulates the production of TNFα [35] and IL-1β [36], the latter being assessed in the monocyte-derived macrophage assay described here. The lack of pyrogen contamination, as determined by the MAT, is further confirmed by the absence of IL-1β generated in response to the nano(bio)materials by MDMs, only present following challenge with LPS. The incorporation of orthogonal measurements of immune cell responses to nano(bio)materials gives a more holistic view of possible issues with application of those materials in human systems [37].

An outcome for this work was to determine the potential for nano(bio)materials to induce cytokines and chemokines from M1- and M2-like macrophages, differentiated ex vivo from primary human immune cells. A variety of factors surrounding routine isolation methods used in the field have been previously highlighted to impact the quality of MDMs [20]. In the interest of generating broadly comparable data, this further highlights the need for a standardised isolation and differentiation protocol. Further, one must bear in mind that polarisation of this cell type is a continuum and not finite, as is the case in vivo [38]. While the approach described in this work may not account for specific phenotypic differences in macrophages resident in particular tissues, e.g., lung macrophages and liver Kupfer cells, etc. [39], it does provide a general surrogate for primary isolated macrophages suitable for the testing of macrophage responses to nano(bio)materials, particularly human samples.

No specific impact to cytokine secretion was observed in response to the NBMs tested under the conditions described here. One must consider that not all cytokines/chemokines are represented by this work, however. The culture supernatants may be retained for further biochemical analysis. Beyond this, the in vivo effects of nano(bio)materials on these and related systems is inevitably more complex, such as the involvement in other mechanisms including CARPA due to perivascular rapid uptake of NBMs [40]. The validity of testing in cell subsets stands to provide an initial indication with good in vitro/in vivo extrapolation [41].

As macrophages derived from primary samples were used in this work, there exists the potential for inter-individual variability in response to positive controls and treatments [42,43,44]. This reality is a double-edged sword whereby the responses generated are more representative of the potential in vivo outcomes [41] and are superior to immortalised cell lines, such as THP1 cells, that will only relate to interactions and responses in a homogeneous manner [18], while increasing complexity in data interpretation when working with relatively low donor numbers. As such, careful consideration must be given to the required output and how it relates to the determination of compatibility of the tested NBMs. In summary, the data described here show that the initial SOP for the differentiation of monocytes for monocyte-derived macrophages holds promise. However, it is clear that there is more work to be done to understand the influence of inter-individual variability on the differentiation of these cells.

## 4. Materials and Methods

### 4.1. Materials

PyroMAT^®^ kit, PyroMAT^®^ cells, PyroDetect^®^ endotoxin standard, non-endotoxin pyrogen control, and Ficoll-Paque premium were purchased from Sigma-Aldrich (Dorset, UK). MACS LS columns, CD14 microbeads (human), MACS BSA stock solution, and autoMACS rinsing solution were purchased from Miltenyi Biotec GmbH (Bergisch Gladbach, Germany). Vacutainers anti-coagulated with Li-heparin were purchased from BD (Swindon, UK). RPMI 1640, foetal bovine serum (FBS), Hank’s balanced salt solution (HBSS), and cell dissociation buffer (enzyme-free, PBS) were purchased from Thermo Fisher (Cheshire, UK). Human M1 macrophage differentiation kit, human M2 macrophage differentiation kit, and Luminex human discovery assay (4-plex, IL-10, IL-12, IL-1β, and IL-1ra) were purchased from Bio-Techne Ltd. (Abingdon, UK) or ThermoFisher Scientific (Waltham, MA, USA, catalogue numbers 88-7126-88, 88-7106-88, and 88-7261-88).

### 4.2. Nano(bio)Materials

LipImage™ 815 [45] was tested as a model liposomal nanomaterial. Two variants of a polymeric nanocarrier composed of the poly(alkyl cyanoacrylate) (PACA) polymer poly(2-ethylbutyl cyanoacrylate) (PEBCA) were tested, one empty and one loaded with cabazitaxel, designated PACA and PACA-CBZ, respectively. Results of their respective characterisation have been described previously by Åslund et al. [22].

#### 4.2.1. LipImage™ 815 Synthesis and Characterisation

Batches of LipImage™ 815 were prepared by high pressure homogenization. The lipid phase comprised 19.125 g of soybean oil, 6.375 g of Suppocire™ NB, 4.875 g of lecithin, and 150 mg of IR-870 oleyl (molar mass: 986.29 g/moL), which was synthesised as previously described [45]. The aqueous phase comprised 25.875 g of Myrj™ S40 and 110 mL NaCl 154 mM. Mixture of lipid and aqueous phases were pre-emulsified using a mechanical disperser (Ultra-T25 Digital Turrax, IKA, Oxford, UK) operated at 15,000 rpm for 5 min. The emulsion was then processed with a High-Pressure Homogenizer (Panda Plus 2000, GEA Niro Soavi, Parma, Italy) operated for 16 cycles with a total pressure of 1250 bars, the pressure of the second stage chamber and the cooling system being set at 50 bars and 30 °C, respectively. Then, 200 g batches of particles were purified by 5 µm filtration followed by tangential flow filtration (Labscale TFF system, Millipore, Darmstadt, Germany) against NaCl 154 mM through a Pellicon XL Biomax™ cassette (Merck, Darmstadt, Germany) operated at a trans-membrane pressure of 14 psi at a flow rate of 2 mL/min. The nanoparticle dispersion was adjusted to a concentration of 100 mg/mL and filtered through a 0.22 µm Millipore membrane for sterilization before storage and use.

Dynamic light scattering (DLS) was used to determine the particle hydrodynamic diameter and zeta potential (Zeta Sizer Nano ZS, Malvern Instrument, Orsay, France). Particle dispersions were diluted to 2 mg/mL of lipids in 0.22 µm filtered 0.1 × PBS and transferred in Zeta Sizer Nano cells (Malvern Panalytical, Malvern, UK) before each measurement, performed in triplicate. Results (Z-average diameter, dispersity index, zeta potential) were expressed as mean and standard deviation of three independent measurements performed at 25 °C. The encapsulation efficiency and payload of IR780-oleyl dye in the LipImage™ 815 were determined by high-performance liquid chromatography (HPLC WATERS Alliance 2695/Fluorescence 2475 detector) and compared with a calibration curve established from the reference fluorophore IR780-Oleyl alone, as previously described [46]. The theoretical amount of IR780-Oleyl encapsulated in a batch of LipImage™ 815 at 100 mg/mL lipid nanoparticles is 266 µM.

#### 4.2.2. PACA Synthesis and Characterisation

PACA nanoparticles were synthesised under aseptic conditions at SINTEF by mini-emulsion polymerization. Prior to synthesis, all solutions were sterile filtered, and all equipment was autoclaved. An oil phase consisting of poly(ethylbutyl cyanoacrylate) (PEBCA) (Cuantum Medical Cosmetics, Barcelona, Spain) containing 2 wt.% Miglyol 812 (Cremer OLEO, Hamburg, Germany) and 10 wt.% vanillin was prepared. For drug-loaded particles, 12 wt.% CBZ (BioChemPartner, Shanghai, China) was added to the oil phase, and only 2 wt.% vanillin was used. For dye-loaded particles, either 0.4 wt.% IR-780-Oleyl (custom synthesis at CEA LETI) or NR668 (modified Nile Red, custom synthesis at SINTEF [47]) was added to the oil phase.

An aqueous phase consisting of 0.1 M HCl containing the two PEG stabilisers Brij^®^L23 (Sigma-Aldrich, St. Louis, MO, USA) and Kolliphor^®^HS15 (Sigma-Aldrich, St. Louis, MO, USA), 5 wt.% of each was added to the oil phase. The water and oil phases were mixed and immediately sonicated for 3 min on ice (6 × 30 s intervals, 60% amplitude, Branson Ultrasonics digital sonifier). The solution was rotated (15 rpm) at room temperature overnight. The pH was then adjusted to 5.0 to allow further polymerisation for 5 h at room temperature. The dispersions were dialyzed (Spectra/Por dialysis membrane MWCO 100,000 Da) against 1 mM HCl to remove unreacted PEG. The size (Z-average), polydispersity index (PDI), and zeta potential of the NPs in phosphate buffer (10 mM, pH 7.0) were measured by DLS and laser Doppler micro-electrophoresis using a Zetasizer Nano ZS (Malvern Instruments, Malvern, UK).

To calculate the amount of encapsulated drug, the drug was extracted from the particles by dissolving them in acetone (1:10), and quantified by liquid chromatography coupled to mass spectrometry (LC-MS/MS) using an Agilent 1290 HPLC system coupled to an Agilent 6490 triple quadrupole mass spectrometer (Agilent Technologies, Santa Clara, CA, USA).

### 4.3. Monocyte Activation Test

The monocyte activation test based on the PyroMAT^®^ system was performed following the manufacturer’s instruction.

#### 4.3.1. Preparation of Standards, Controls, and Test Materials

A seven-point endotoxin standard curve was prepared in pyrogen-free and non-endotoxin absorbing glass tubes. The standard endotoxin solutions were prepared from the PyroDetect^®^ endotoxin standard stock solution at 2000 EU/mL. The endotoxin standard was diluted in endotoxin-free water to concentrations of 0.0125, 0.025, 0.05, 0.1, 0.2, 0.4, and 0.8 EU/mL. An additional 20 EU/mL dilution was prepared to evaluate spike recovery.

Heat-killed Staphylococcus aureus (HKSA) was included as a positive control to assess the detection of non-endotoxin pyrogens by the system. The stock (1000× concentration) solution was diluted to 100× for spiking of NBMs and 1× to evaluate the spike recovery. LipImage™ 815, PACA, and PACA-CBZ were prepared to final concentrations of 0.4, 2, and 10 µg/mL with endotoxin-free water, using endotoxin-free glass tubes.

#### 4.3.2. PyroMAT^®^ Cell Incubation/Stimulation of IL-6 Production

To appropriate wells, the standard curve, HSKA, NBMs, and NBMs spiked with either endotoxin or HSKA were loaded. Endotoxin-free water was included as a blank. Four replicates were prepared for each condition. The PyroMAT^®^ cells (Mono-Mac-6 human monocytic cell line) were suspended in warmed RPMI medium, 200 µL of which was dispensed per well. The cell culture plate was incubated for 22 h at 37 °C, in humidified conditions.

#### 4.3.3. IL-6 ELISA Procedure

All components were equilibrated to room temperature. Then, 100 µL/well of assay diluent was loaded to the ELISA plate, and an equal volume of cell supernatant added and mixed. Following 2 h incubation, the contents from each well were aspirated and washed four times with excess wash buffer. Then, 200 µL/well of IL-6 conjugate was added to each well, incubated for 2 h, and washed a further four times. Following this, 200 µL/well of substrate solution was added and incubated for 20 min at room temperature, protected from light. Then, 50 µL/well of stop solution was added and mixed thoroughly by pipetting to ensure colour homogenization prior to absorbance measurement using a CLARIOstar plate reader (BMG Labtech, Ortenberg, Germany) at 450 nm with reference wavelength of 630 nm.

### 4.4. Monocyte Isolation and Differentiation to M1/M2 Macrophages

#### 4.4.1. Blood Collection and Lymphocyte Isolation

Blood was collected from healthy volunteers via venepuncture, into vacutainers anti-coagulated with Li-heparin, under ethical approval granted by the University of Liverpool Committee in Research Ethics (Ref: RETH000563, approved 1 June 2012). Informed consent was given by the volunteers for the use of whole blood in subsequent assays. Work at the JRC was carried out on blood samples from health volunteers following informed consent (DPR-EC-01935) and in compliance with the Human Tissue Authority Code of Practice. The use, by RIVM, of buffy coats for the current study was authorised by Sanguin (Amsterdam, The Netherlands) under agreement NVT0243.02. All samples were anonymised. Isolation of peripheral blood mononuclear cells (PBMC) was performed as previously described [24]. Blood was layered over Ficoll-Paque separation medium at a 2:1 ratio and centrifuged for 30 min at 900× *g*, 18–20 °C, without brake. The PBMC layer was transferred to a fresh universal tube and washed three times in an excess of HBSS, and centrifuged for 10 min at 400× *g*, at 18–20 °C. PBMCs were suspended in RPMI-1640 supplemented 10% with FBS, counted, and viability checked to be at least 90%.

#### 4.4.2. CD14^+^ Monocyte Magnetic Labelling and Isolation

Magnetic isolation of CD14^+^ monocytes was performed following the manufacturer’s protocol (Ref. 140-000-067.07) as described previously [24]. Magnetic labelling and isolation buffer was prepared by diluting MACS BSA stock solution 1:20 with autoMACS rinsing solution. The cell suspension was centrifuged for 10 min at 300× *g*, and the cell pellet was resuspended in 80 µL of buffer per 10^7^ total cells, to which 20 µL of CD14 microbeads per 10^7^ total cells was added, mixed, and incubated for 15 min, at 2–8 °C. The cells were washed by adding 1–2 mL of buffer per 10^7^ cells and centrifuged 10 min at 300× *g*, and resuspended up to 10^8^ cells in 500 µL of buffer. The cell suspension was applied to primed LS columns in the magnetic field of the MACS Separator and washed three times with buffer, discarding the effluent. The labelled cells were collected by flushing outside the magnetic field into a fresh universal tube.

#### 4.4.3. M1/M2 Macrophage Differentiation

Isolated CD14^+^ monocytes were differentiated to either M1 macrophages using GM-CSF or M2 macrophages using M-CSF, supplied in the human M1 macrophage differentiation kit and human M2 macrophage differentiation kit, respectively. Human M1 and M2 macrophage differentiation media were prepared according to the manufacturer’s protocol (Ref. 726381.2 and 726382.2, respectively). The CD14^+^ monocytes were seeded in 6-well plates, at 4 mL/well, at 2 × 10^6^ cells/mL in either human M1 or M2 macrophage differentiation media, and incubated for three days (37 °C, 5% CO_2_, humidified). On day three, 2 mL per well was removed and replenished with an equal volume of fresh differentiation media, and incubated for an additional three days. On day six, culture supernatants were collected and centrifuged for 10 min at 300× *g*, retaining cell pellets. Then, 2 mL of enzyme-free dissociation buffer was added to each well and incubated at 37 °C for 30 min. Adherent cells were then detached by gently pipetting. Cell suspensions were combined with their respective non-adherent pellets and further centrifuged. M1 and M2 macrophages were resuspended in RPMI 1640 supplemented with 10% FBS and counted.

### 4.5. Macrophage Exposure to NBMs, and Subsequent Cytokine Analysis

M1 and M2 macrophages were seeded at a density of 1 × 10^6^ viable cells/mL and were exposed to negative control (PBS), positive control (20 ng/mL LPS), or NBMs at a final concentration of 10 µg/mL for a period of 24 h (37 °C, 5% CO_2_, humidified). Culture supernatants were collected in 1.5 mL centrifuge tubes, spun in a microcentrifuge at maximum speed (17,000× *g*) for 5 min to remove any non-adherent cells, and transferred into fresh tubes.

#### Cytokine Quantification

Determination of cytokine concentrations was carried out using multiplex cytokine assays conducted using the Bio-Plex 200 Luminex system (Bio-Rad, Hercules, CA, USA). At UOL, cytokines IL-1β, IL-10, IL-1ra, and IL-12 were measured, and at RIVM IL-12p70, IL-10 and IL-1β. Briefly, antibody-coated magnetic beads (50 µL) were loaded to a 96-well plate, and an equal volume of cell culture supernatant fractions were added, alongside multiplexed standard curves for relevant cytokines. Samples were incubated on a plate shaker for 2 h at room temperature. The plate was washed with wash buffer three times using an automated magnetic plate washer prior to the addition of biotinylated detection antibody (50 µL) and then incubated on a plate shaker for 1 h at room temperature. The plate was again washed three times prior to the addition of streptavidin-PE (50 µL) and incubation on a plate shaker for 30 min. The plate was then washed three times, and assay buffer (100 µL) was added to wells. The plate was analysed on a Bioplex 200 analyser using the recommended gating settings.

### 4.6. Statistics

Graphs and statistical analyses were performed with GraphPad Prism 7 and/or Microsoft Excel 2016. All data are displayed as an average ± standard deviation. Differences between controls and treatments were evaluated by *t*-test. Statistical significance was considered at *p* < 0.05.

## Data Availability

The datasets generated and/or analysed during the current study are available from the corresponding author on reasonable request.

## Abbreviations

The following abbreviations are used in this manuscript:

CARPA	Complement activation-related pseudoallergy
CBZ	Cabazitaxel
CD14	Cluster of differentiation 14
ELISA	Enzyme-linked immunosorbent assay
EU	Endotoxin units
FBS	Fetal bovine serum
FDA	Food and Drug Administration
GM-CSF	Granulocyte-macrophage colony-stimulating factor
GMP	Good manufacturing practice
HBSS	Hank’s balanced salt solution
HIV	Human immunodeficiency virus
HSKA	Heat-killed Staphylococcus aureus
IL	Interleukin
LAL	Limulus amoebocyte lysate
LPS	Lipopolysaccharide
MAT	Monocyte-activation test
MDM	Monocyte-derived macrophage
M-CSF	Macrophage colony-stimulating factor
NBM	Nano(bio)materials
PACA	Poly(alkyl cyanoacrylate)
PBMC	Peripheral blood mononuclear cell
PEBCA	Poly(ethylbutyl cyanoacrylate)
PE	Phycoerythrin
PEG	Polyethylene glycol
PBS	Phosphate-buffered saline
REFINE	Regulatory Science Framework for Nano(bio)material-based Medical Products and Devices
RPMI-1640	Roswell Park Memorial Institute 1640 Medium
RPT	Rabbit pyrogen test
SOP	Standard operating procedure
TNFα	Tumour necrosis factor alpha
